# Transactions of the Section of Obstetrics and Diseases of Women of the American Medical Association at the Fifty-Seventh Annual Meeting, Held at Boston, June 5-8, 1906

**Published:** 1907-04

**Authors:** 


**Transactions of the Section of Obstetrics and Diseases of Women** of the American Medical Association at the fifty-seventh annual meeting, held at Boston, June 5-8, 1906.

The transactions of this section at the Boston meeting makes an interesting volume which is presented in a readable form. The presswork is beyond mere commendation; it is exquisite in its perfection. The illustrations bring the volume close to the de luxe line. Every paper reprinted is of especial interest.

